# Supplementary material for: Transcriptome Analysis of Ochratoxin A-Induced Apoptosis in Differentiated Caco-2 Cells
Source: Toxins (Basel). 2019 Dec 31;12(1):23. doi: 10.3390/toxins12010023 (PMC7020595; doi:10.3390/toxins12010023)
Supplement: Supplementary file 1 [file toxins-12-00023-s001.pdf]

Supplementary Materials: Transcriptome Analysis of Ochratoxin A-Induced Apoptosis in Differentiated Caco-2 Cells

Yang Xue, Yanan Gao, Qiaoyan Yan, Xiaoyu Bao, Shengguo Zhao, Jiaqi Wang and Nan Zheng

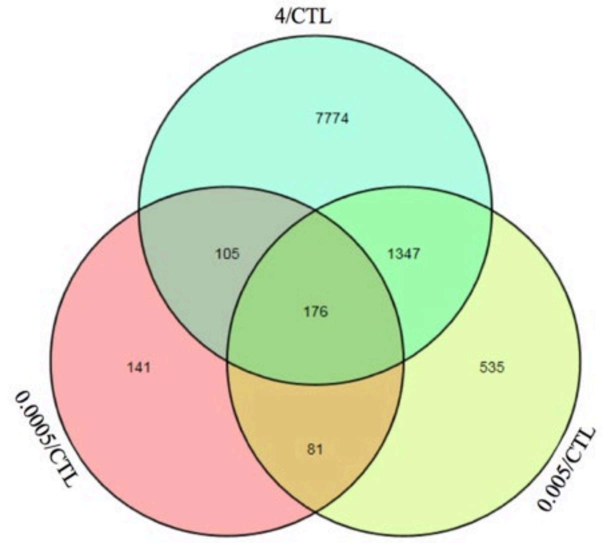

Figure S1. Venn diagram depicting the DEGs common to different dose of OTA exposure.

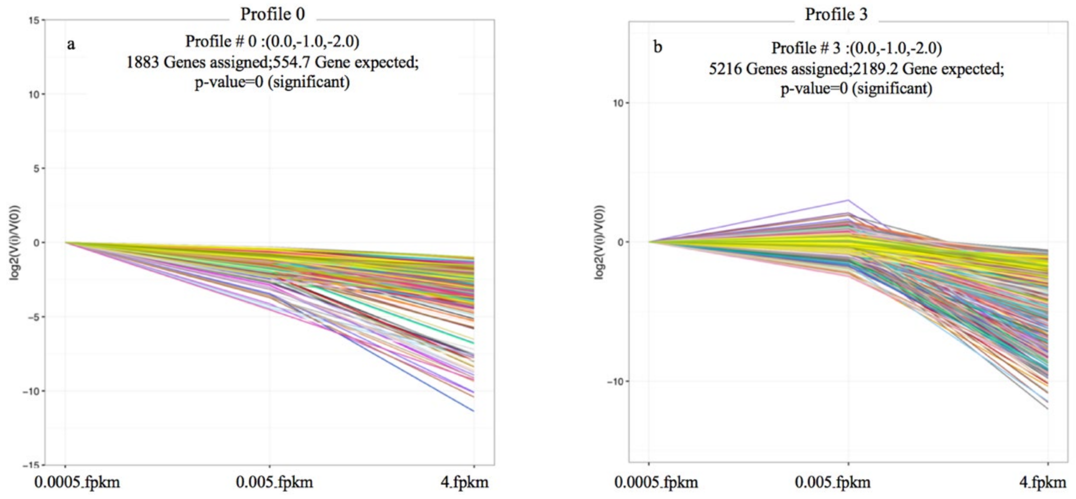

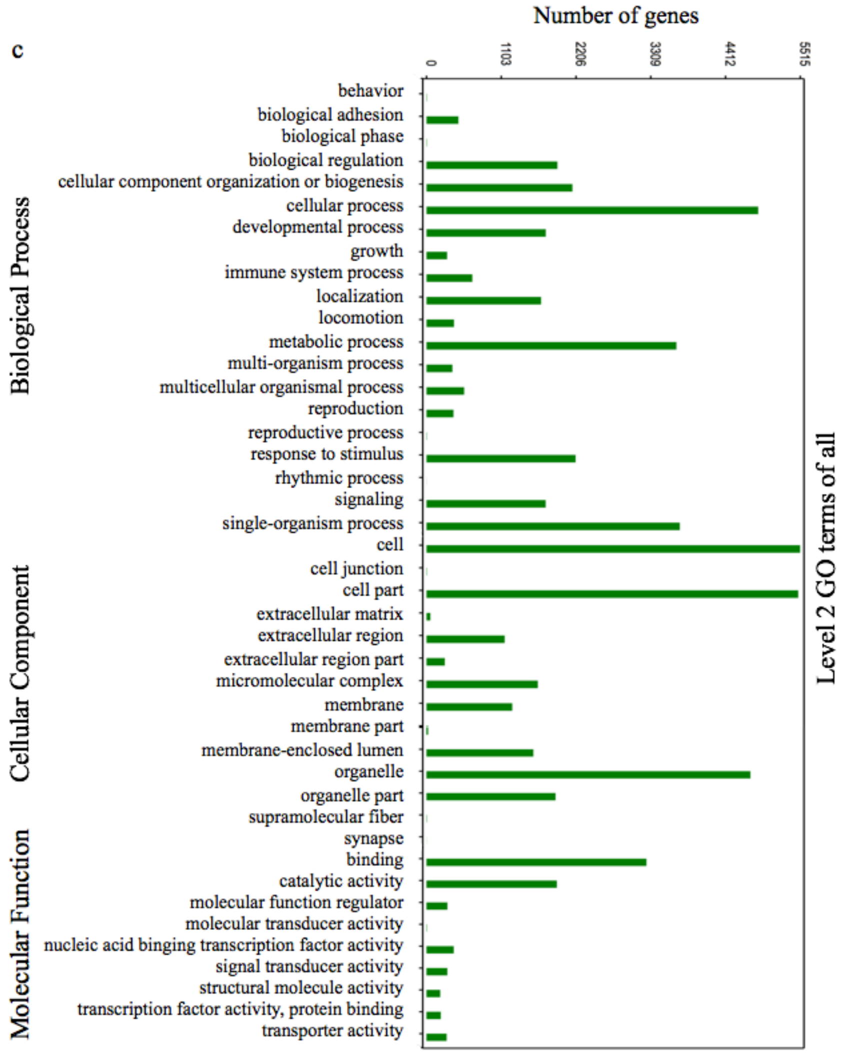

Figure S2. DEGs expression profiles in ochratoxin A (OTA) treated differentiated Caco-2 cells. Concentrations of 0.0005, 0.005, and 4 μg/mL OTA were used. Profile 0 (**a**) and Profile 3 (**b**) indicating down-regulating trend during 48 h of OTA treatment, and their GO annotation (**c**).

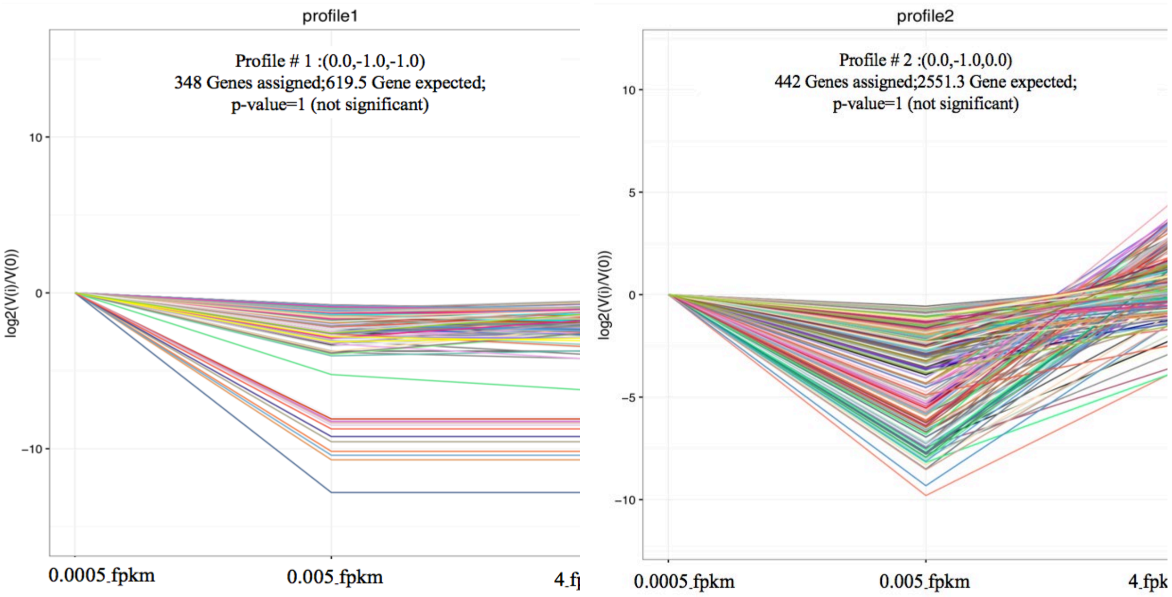

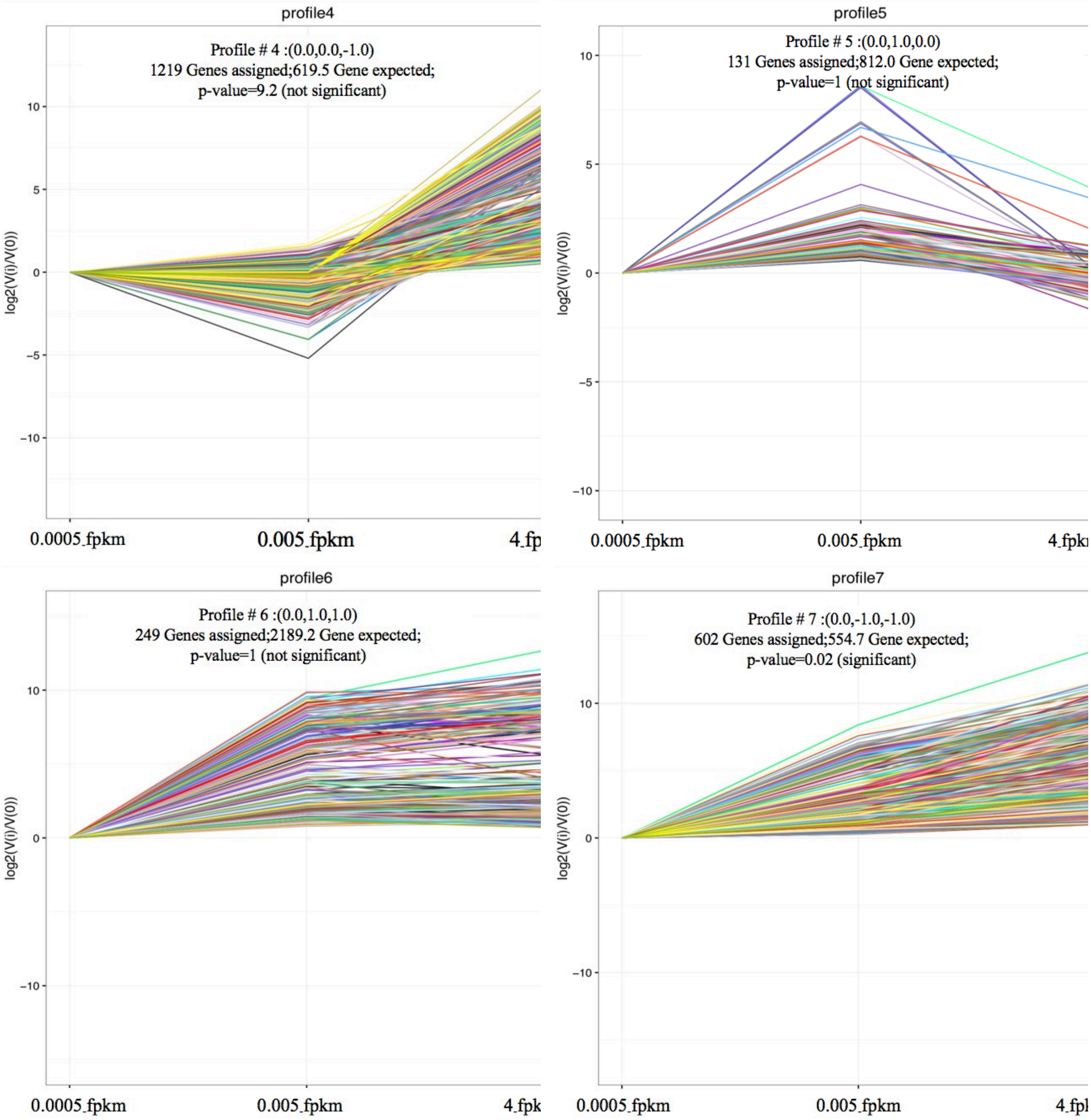

Figure S3. DEGs expression profile in the OTA treated differentiated Caco-2 cells. Concentrations of OTA used is 0.0005, 0.005, and 4 μg/mL. Except for two significantly down-regulated profiles(profile 0 and 3), there are six different expression profiles (profile 1, 2, 4, 5, 6, 7).
